# LC-MS Based Platform Simplifies Access to Metabolomics for Peroxisomal Disorders

**DOI:** 10.3390/metabo11060347

**Published:** 2021-05-29

**Authors:** Henry Gerd Klemp, Matthias Kettwig, Frank Streit, Jutta Gärtner, Hendrik Rosewich, Ralph Krätzner

**Affiliations:** 1 Department of Pediatrics and Adolescent Medicine, University Medical Center Göttingen, Robert-Koch-Str. 40, 37075 Göttingen, Germany; henrygerd.klemp@stud.uni-goettingen.de (H.G.K.); matthias.kettwig@med.uni-goettingen.de (M.K.); kinderklinik.sekretariat@med.uni-goettingen.de (J.G.); 2 Department of Clinical Chemistry, University Medical Center Göttingen, Robert-Koch-Str. 40, 37075 Göttingen, Germany; frank.streit@med.uni-goettingen.de

**Keywords:** peroxisome biogenesis disorder, Zellweger syndrome spectrum, metabolomics, membrane lipids, biomarker, biocrates, AbsoluteIDQ p180, PEX1, ABCD1, D-BPIII, VLCFA, PC ae 36:4

## Abstract

Peroxisomes are central hubs for cell metabolism and their dysfunction is linked to devastating human disorders, such as peroxisomal biogenesis disorders and single peroxisomal enzyme/protein deficiencies. For decades, biochemical diagnostics have been carried out using classical markers such as very long-chain fatty acids (VLCFA), which can be inconspicuous in milder and atypical cases. Holistic metabolomics studies revealed several potentially new biomarkers for peroxisomal disorders for advanced laboratory diagnostics including atypical cases. However, establishing these new markers is a major challenge in routine diagnostic laboratories. We therefore investigated whether the commercially available AbsoluteIDQ p180 kit (Biocrates Lifesciences), which utilizes flow injection and liquid chromatography mass spectrometry, may be used to reproduce some key results from previous global metabolomics studies. We applied it to serum samples from patients with mutations in peroxisomal target genes *PEX1*, *ABCD1*, and the *HSD17B4* gene. Here we found various changes in sphingomyelins and lysophosphatidylcholines. In conclusion, this kit can be used to carry out extended diagnostics for peroxisomal disorders in routine laboratories, even without access to a metabolomics unit.

## 1. Introduction

Peroxisomes are central hubs for lipid metabolism inside cells [1,2,3]. They are the exclusive organelle for catabolism of very long chain fatty acids (C > 22 atoms; VLCFAs) and participate in a wide range of lipid biosyntheses such as for plasmalogens [4]. Furthermore, peroxisomes take part in the biosynthesis of bile acids and degradation of D-amino acids as well as pristanic and phytanic acid [5,6].

Disorders of peroxisomal biogenesis are caused by mutations in *PEX* genes, follow an autosomal recessive inheritance, and are characterized by complex clinical phenotypes typically involving the brain, liver, and kidneys. The group of peroxisomal biogenesis defects leading to Zellweger syndrome spectrum disorders (ZSS) show a broad clinical range from mild to severe forms with overlapping symptoms. Special morphological features can be present like facial dysmorphism or bone abnormalities, including calcific stippling (chondrodysplasia punctata), especially in the knees and hips. [7,8]. Neurological symptoms can range from attention-deficits and behavioral symptoms to dementia, poor motor development, and epilepsy.

Owing to the centrality of peroxisomes in VLCFA lipid metabolism [9], the main marker for detection of ZSS is the total amount of lignoceric acid (24-carbon fatty acid) or cerotic acid (26-carbon fatty acid) analyzed by gas chromatography mass spectrometry (GC-MS). VLCFA levels are usually stated as ratio to a non-VLCFA, such as behenic acid (22-carbon fatty acid), presenting more nutrition independent levels then VLCFAs alone. As peroxisomes play an important part in the ether-phospholipid biosynthesis, plasma-logens are another important key metabolite. Additionally, other substrates only handled by the peroxisomes such as phytanic acid and pristanic acids are analyzed. Finally, pipecolic acid and bile acids can be determined as supplementary markers.

As mentioned, these analytes are determined using GC-MS [10,11]. Though GC-MS provides very stable results for diagnostics, it often requires perilous and error-prone derivatization procedures and provides low analytical speed. Due to low prevalence of per-oxisomal disorders, analytical speed is of lower importance to diagnostics. For scientific research, however, it is highly beneficial—particularly for larger screening studies of potential treatments.

Over the recent years, a number of milder and atypical cases of ZSS were reported, where classical diagnostic peroxisomal key metabolites like VLCFA and plasmalogens revealed minor or even no abnormalities [9,12,13]. These findings suggest that there are mild cases, which cannot be diagnosed biochemically via the classical peroxisomal lead metabolites and thus cannot be genetically confirmed.

It is therefore necessary to identify new biomarkers for ZSS patients, which can reliably identify both the classical and mild variants and already better distinguish the different phenotypes biochemically. In this context, several untargeted metabolic profiling studies with blood samples and primary cultured human fibroblast of affected ZSS patients were carried out, indicating the existence of further biomarkers for a more differentiated laboratory diagnostic view on that disease group.

In an untargeted metabolomics study with 19 subjects classified as mild-to-moderate ZSS, all nine further examined sphingomyelins were shown to be dramatically reduced in affected patients [14]. A subsequent screening of more than thousand patient samples excluding known ZSS samples revealed only 21 samples with reduced values for multiple sphingomyelins. In one of these samples, also elevated VLCFA levels were detected subsequently, and the patient was finally diagnosed with D-bifunctional protein deficiency type 3 (D-BPIII), a disorder of peroxisomal β-oxidation. This study highlights the role of peroxisomes in sphingomyelin metabolism and of sphingomyelins as potential peroxisomal biomarkers.

In an untargeted lipidomics approach on samples of patients with peroxisomal disorders, 1365 distinct phospholipids, cholesterol esters, and di- and triglyceride species were annotated [15]. In this study, levels of different species of phosphatidylcholines, lysophosphatidylcholines, ether phospholipids, sphingomyelins and other lipids were found to be significantly altered in affected patients. In addition, among the ether-lipids, also plasmalogens were detectable in the plasma samples of healthy individuals and missing in patients with peroxisomal biogenesis disorders. Similar results were obtained from untargeted metabolomics studies with fibroblasts [16,17].

While these new markers may be of promising diagnostic potential, an obvious disadvantage lies in the elaborated and time-consuming workflow of processes associated with untargeted metabolomics approaches. In addition, a high grade of technical and scientific expertise is needed that is not easily available in routine diagnostic mass spectrometry laboratories.

To overcome these problems, we applied a commercially available kit for targeted metabolomics in a study with patient samples associated with ZSS (*PEX1*-mutation), X-linked adrenoleukodystrophy (X-ALD, *ABCD1*-mutation) and, in a very small subgroup, D-bifunctional protein deficiency type III (D-BPIII, *HSD17B4*-mutation). This kit provides consumables, measurement, as well as data analysis methods for the rapid establishment of a metabolomics pipeline. After setup the user is almost instantly able to quantify up to 187 metabolites (40 acyl-carnitines, 15 sphingolipids, 76 phosphatidylcholines, 15 lysophosphatidylcholines, 42 amino acids, and other biogenic amines, Available online at: https://www.biocrates.com/products/research-products/absoluteidq-p180-kit, accessed on 20 May 2021). Since its release it has been widely adapted in many laboratories and as opposed to intra-laboratory pipelines, validated in a multi-centered ring trial and concepted in accordance with FDA and EMA recommendations [18].

In this study, we tested whether this targeted metabolomics kit is able reproduce key findings from previous studies or even discover new potential markers in peroxisomal disorders.

## 2. Results

### 2.1. 143 Metabolites Analyzed from Plasma Samples

Assessing suitability of the AbsoluteIDQ p180-kit from Biocrates for analysis of the metabolism of peroxisomal disorders, we compared serum samples of 17 ZSS patients with *PEX1* mutations to a non-affected age-matched control group, as well as 36 X-ALD patients with mutations in the *ABCD1* gene and two DPBIII patients with mutations in the *HSD17B4* gene. Of the 187 metabolites analyzed by the kit, 143 metabolites with concentrations higher than the limit of quantification were used for further analysis.

### 2.2. ZSS Patients Show Decreased Acyl-Ether-Linked Phospholipids

**Figure 1:**
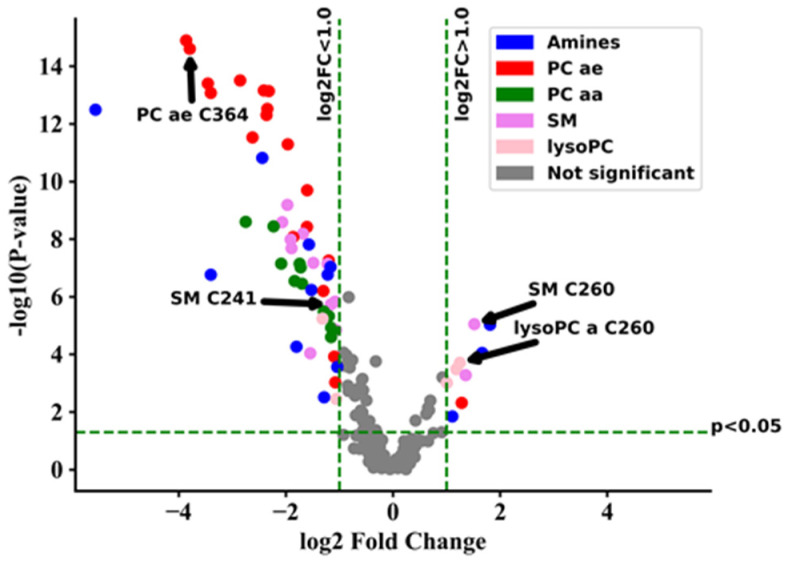
Volcano-plot showing metabolic changes of Zellweger syndrome spectrum patients (ZSS/*PEX1*) in serum compared to control group. Components with a raw *p*-value < 0.05 and log2 fold change (log2FC) > 1.0 are displayed in color. lysoPC: lysophosphatidylcholine; PC aa: phosphatidylcholines with two acyl-linkages; PC ae: phosphatidylcholine with one acyl and one ether linkage; SM: sphingomyelin.

Major changes in the metabolome of ZSS patients compared to controls included the decrease of acyl-ethyl-linked phospholipids (including plasmalogens, PC ae, e.g., PC ae C36:4, C40:6,) as visualized in the volcano-plot (Figure 1, Table A1).

**Figure 2:**
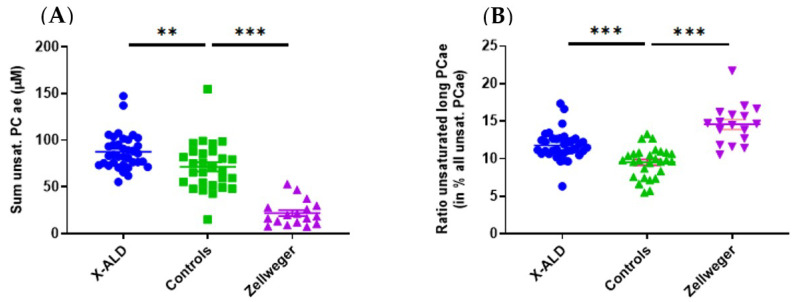
(**A**) Total sum of polyunsaturated acyl-ethyl-phosphatidylcholines (PC ae/plasmalogen candidates) is lower in Zellweger syndrome spectrum patients (ZSS, *PEX1*) and higher in X-linked adrenoleukodystrophy (X-ALD, *ABCD1*) patients than in controls. (**B**) Unsaturated (very) long chain PC ae (total C > 42) are enriched compared to total polyunsaturated PC ae in ZSS and X-ALD patients. Mean ± SEM, *n* = 36 (X-ALD/*ABCD1*), 28 (Controls), 17 (ZSS/*PEX1*). ** *p* < 0.01, *** *p* < 0.001 after one-way ANOVA multiple comparisons analysis, Tukey correction.

**Figure A1:**
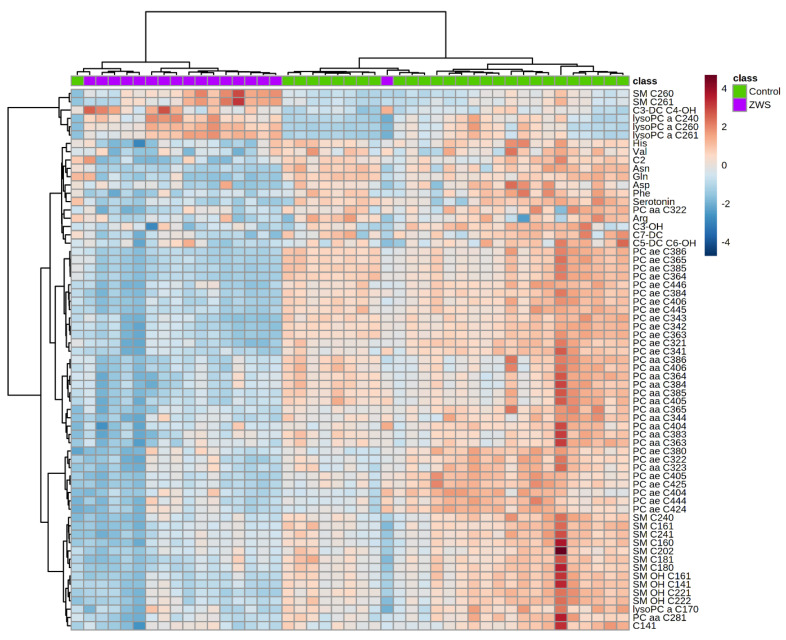
Heatmap illustrating clustering of Zellweger syndrome spectrum patients (ZSS) and controls. Serum from patients analyzed using the Biocrates AbsoluteIDQ p180 kit. Only the 65 components with highest *p*-value by Student’s *t*-test are shown. Euclidean distance and Ward clustering were used. Normalized data was used and colormaps were auto scaled. *n* = 28 (Controls), 17 (ZSS/*PEX1*). C: Acyl-Carnitine; lysoPC: lysophosphatidylcholine; PC aa: phosphatidylcholines with 2 acyl-linkages; PC ae: phosphatidylcholine with one acyl and one ether linkage; SM: sphingomyelin.

Using the metabolites provided by the kit, a separated clustering of controls and ZSS patients was achieved (Figure A1). In previous classical metabolomics analyses, acyl-ethyl-linked phospholipids such as PC ae C36:4, C40:6, C44:5 were similarly decreased in ZSS patients [15]. That especially applies for the group of polyunsaturated PC ae (including plasmalogens), which were lower in ZSS patients (22.11 µM ± 3.27) and slightly higher in X-ALD patients (87.67 µM ± 3.16) than in controls (71.55 µM ± 4.92; Figure 2).

The measurement of ether-lipids by GC-MS is a standard method for the biochemical diagnosis of ZSS. Thus, we also examined C16-diacetal and C18-diacetal, two classical GC-MS plasmalogen markers. Interestingly in four cases, C18-diacetal was inside normal reference ranges and in three cases also C16-diacetal presented normally (Table A2). Yet these patients presented with PC ae C36:4 levels clearly in the pathological range. This may point to the utility of this marker in diagnosing ZSS.

For patients that were above the detection limit for C18-diacetal, we found a correlation to the ether-phosphocholines PC ae C36:0 and PC ae C36:4. PC ae C36:0 and PC ae C36:4, most likely possess an ethyl-linked C18 fatty acid. Albeit only a very small subgroup of five patients could be included and results should be confirmed in larger studies.

Classically, analysis of ether lipids with GC-MS can only be done from freshly prepared erythrocyte membranes and not from serum [19]. As erythrocyte membranes are not stable over long periods and not stored in biobanks, a retrospective analysis of ether lipids in patients after longer time periods is commonly not possible.

Contrary to older studies based on GC-MS [19], as well our studies as also the lipidomics study of Herzog et al. [16] could show stable changes of ether-lipids in plasma samples, not only in erythrocyte membranes. As some of the samples had a storage time of over 10 years, also long-time stability of those analytes appears to be superior. To our knowledge, this is the first study to demonstrate the use of a FIA-MS based method for the study of ether-phospholipids in plasma in ZSS patients.

### 2.3. VLCFA-Linked Membrane Lipid Levels Are Increased in Both Peroxisomal Disorders, but Overall Sphingomyelin Levels Are Only Decreased in ZSS Patients

Plasma levels of most sphingomyelin species (such as SM C24:1, C24:0, C16:0, C16:1, C18:0, C18:1; Table A3) showed a significant decrease in ZSS patients, however the concentration of VLCFA-linked SM C26:0 was highly increased compared to control samples. 

SM C26:0 levels were also increased in X-ALD patients, but shorter chain sphingomyelins levels were not as reduced as in Zellweger patients. Alterations of sphingomyelin levels have also been found in another lipidomics study [14], especially reporting reduction in SM C16:0, C16:1, C18:1, C22:0, and C24:1of ZSS patients. Yet another study showed accumulations of SM with a fatty acid longer then C > 24:0 in fibroblasts cells from ZSS patients [16], matching our observation of SM C26:0 in plasma.

**Figure 3:**
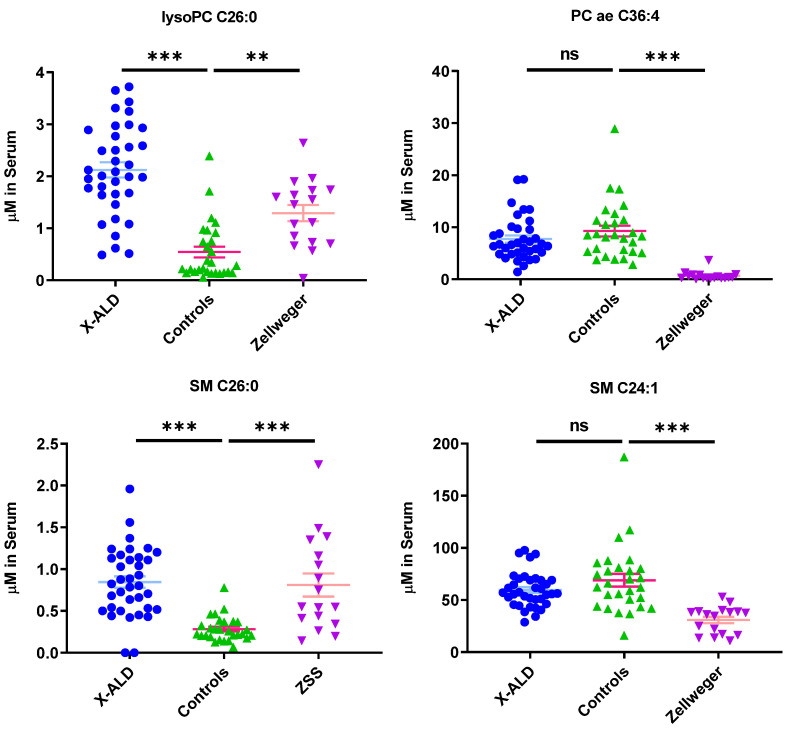
Phospholipids indicate differential phenotype between Zellweger syndrome spectrum patients (ZSS) and X-linked adrenoleukodystrophy (X-ALD) patients, as well as controls. Mean ± SEM, *n* = 36 (X-ALD/*ABCD1*), 28 (Controls), 17 (ZSS/*PEX1*). ns: not significant, ** *p* < 0.01, *** *p* < 0.001 after one-way ANOVA multiple comparisons analysis, Tukey correction. lysoPC: lysophosphatidylcholine; PC aa: phosphatidylcholines with two acyl-linkages; PC ae: phosphatidylcholine with one acyl and one ether linkage; SM: sphingomyelin.

In addition to SM C26:0, lysoPC C26:0 was found to be increased in ZSS and X-ALD patients (Figure 3).

LysoPC C26:0 is used as a marker in newborn screening for X-ALD [20] and is commonly found in other lipidomics studies [14,16]. Increases in plasma concentrations of other phospholipids such as phosphatidylcholines with very long fatty acid chains (C > 42) were described by Herzog et al. in fibroblasts [16], but were not found in our analysis, as only the species up to C42 for PC aa and C44 for PC ae were included. However, if we look specifically for the fraction of PC ae (C > 42) with long chain unsaturated fatty acids in relation to the total amount of PC ae we find an increase in ZSS and X-ALD patients compared to controls (Figure 2).

### 2.4. Peroxisomal Disorders Show Disease Specific Clustering

**Figure 4:**
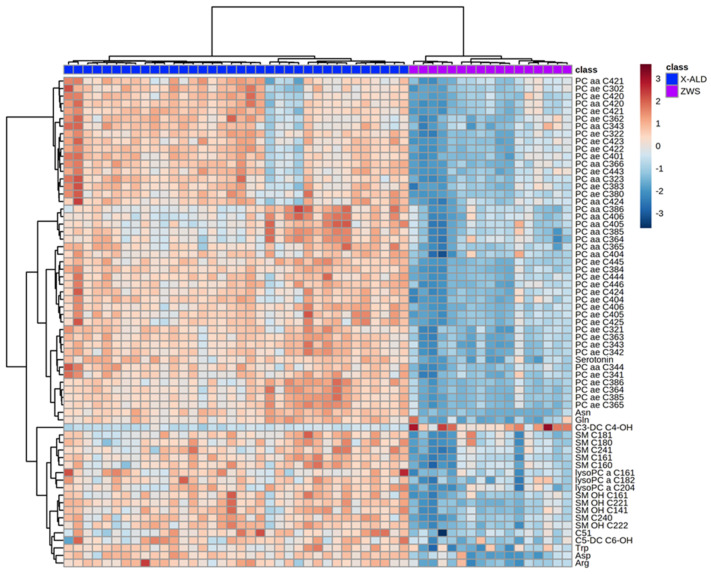
Heatmap illustrating clustering of Zellweger syndrome spectrum patients (ZWS) and X-linked adrenoleukodystrophy (X-ALD) patients. Serum from patients analyzed using the Biocrates AbsoluteIDQ p180 kit. Only the 65 components with highest *p*-value by Student’s *t*-test are shown. Euclidean distance and Ward clustering were used. Normalized data was used and color maps were auto scaled. *n* = 36 (X-ALD/*ABCD1*), *n* = 17 (ZSS/*PEX1*). C: Acyl-Carnitine; lysoPC: lysophosphatidylcholine; PC aa: phosphatidylcholines with two acyl-linkages; PC ae: phosphatidylcholine with one acyl and one ether linkage; SM: sphingomyelin.

By analyzing the top 65 metabolites with the most significant changes in plasma levels between ZSS and X-ALD, it is possible to differentiate between the two diseases on the basis of clustering (Figure 4).

### 2.5. X-ALD Patients Can Be Separated from Controls, but Show a Less Stringent Metabotype

**Figure A2:**
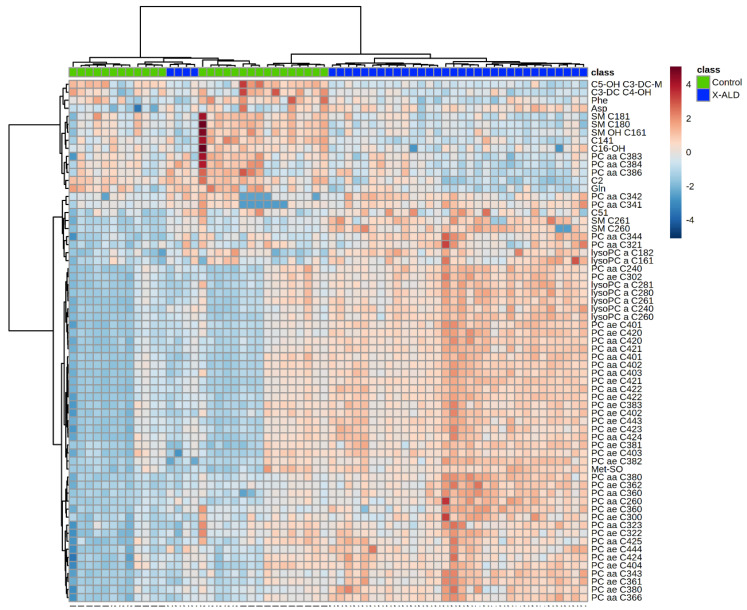
Heatmap illustrating clustering of X-linked adrenoleukodystrophy (X-ALD) patients and controls. Serum from patients analyzed using the Biocrates AbsoluteIDQ p180 kit. Only the 65 components with highest *p*-value by Student’s *t*-test are shown. Euclidean distance and Ward clustering were used. Normalized data was used and colormaps were auto scaled. *n* = 36 (X-ALD/*ABCD1*), 28 controls. C: Acyl-Carnitine; lysoPC: lysophosphatidylcholine; PC aa: phosphatidylcholines with 2 acyl-linkages; PC ae: phosphatidylcholine with one acyl and one ether linkage; SM: sphingomyelin.

Using the metabolites provided by the kit, we were able to separate patients from controls (Figure A2). Among the changes are especially high VLCFA-lysoPCs concentrations in patients (Table A4). However, X-ALD does not lead to a metabolic profile as stringent as in ZSS patients. Even when considering the 65 metabolites with the highest difference for a corresponding clustering, four X-ALD patients could not be differentiated from the control patients.

### 2.6. D-Bifunctional Protein Deficiency Type III Patients Show Alterations of Peroxisomal Markers

Additionally to X-ALD and ZSS, we aspired to gain first insights if the kit may also be able to differentiate another peroxisomal disorder. For this, we analyzed two patients with D-bifunctional protein deficiency type III (D-BPIII), where two samples at different time points for patient 1 were available. Patient 1 showed higher lysoPC C26:0 levels than the control samples, with 1.33 µM and 5.86 µM, respectively. For patient 2 that level was also increased with 3.97 µM compared to 0.54 ± 0.15 µM in controls (controls found in Table A4). Thus, a similar increase in concentration of lysoPC C26:0 as in ZSS and X-ALD was observed.

PC ae C36:4 was slightly decreased in patients compared to controls, with 5.66 µM and 5.32 µM in patient 1, 2.82 µM in patient 2 and 9.28 ± 1.04 µM in controls. 

While a decrease of ether-phospholipids is uncommon for D-BPIII patients, a decrease was also found in GC-MS analysis (Table A5, GC-MS was only acquired once from patient 1). However, in GC-MS analysis, there was also a high number of ZSS patients with comparatively high plasmalogen levels, so that a differentiation between D-BPIII and ZSS patients was not possible. Even so, a decrease of PC ae 36:4 was visible using the kit, the separation of D-BPIII patients and ZSS patients (0.67 ± µM) was considerably greater. This may allow for superior detection sensitivity of abnormalities using PC ae 36:4 as a ZSS biomarker.

SM C24:1 and SM C26:0 showed comparable concentrations as in ZSS with 21.2 µM and 35.5 µM (Patient 1), 32.6 µM (Patient 2) for SM C24:1 and 0.525 µM and 1.13 µM (Patient 1) and 1.08 µM (Patient 2) for SM C26:0.

Summarizing, we found an increase in VLCFA lipids lysoPC C26:0 and SM C26:0, and small decreases in ether-linked membrane lipids, such as PC ae C36:4. Similar results with an increase in lysoPC C26:0 and SM C26:0 and a drop in ether-phospholipids was described by Herzog et al. [17] for D-BPIII in patient fibroblasts.

### 2.7. Summary of Results

Metabolomics analysis using the fast Absolute IDQ p180-kit from Biocrates showed separate metabolic profiles of X-ALD and ZSS patients as well as controls. LysoPC C26:0 and SM C26:0 were elevated in both peroxisomal disorders; however, total plasmalogen candidates (polyunsaturated PC ae) were decreased only in ZSS patients, but slightly increased in X-ALD patients.

## 3. Discussion

Over the recent years, several milder, atypical cases of peroxisomal disorders were reported where classical laboratory markers revealed minor or even no abnormalities. Such highlights the need for additional biomarkers to expand the spectrum of available diagnostic parameters in addition to targeted use of modern genetics [9,13,14]. Promising biomarkers to recognize and more precisely differentiate these milder cases were identified in various global metabolomic studies [14,16]. In routine clinical diagnostic laboratories, resources like equipment, time and specialized knowledge to conduct untargeted metabolomics studies are limited. In addition, untargeted metabolomics studies are not easily comparable between study centers. Thus, we selected to apply a commercially available, international ring-trial validated, kit for targeted metabolomics to overcome those limits. This kit named “AbsoluteIDQ p180-kit” from Biocrates includes multiple analytes of metabolite groups previously identified as potentially new biomarkers for peroxisomal disorders. It was easily established in our laboratory and did not require specific expertise for metabolomics, but only knowledge common to clinical chemistry mass spectrometry facilities. All plasma samples used to evaluate the kit were recruited from an in-house biobank; the patients were already genetically diagnosed as affected by ZSS, X-ALD, or D-BPIII.

Using the kit, we were able to differentiate a group of patients with ZSS from patients with X-ALD. ZSS patients and X-ALD patients showed a divergent lipid pattern in the heatmap. By applying hierarchical clustering, patients were grouped as two uniform groups. Given the set of analyzed metabolites, this is not surprising, as the peroxisomal biogenesis disorders leading to ZSS influence a multitude of pathways compared to the single peroxisomal protein defect causing X-ALD.

On closer inspection of the metabolites having aberrant concentrations, polyunsaturated ether-phosphatidylcholines (PC ae, “total plasmalogen candidates”, especially PC ae C36:4, C40:6) are decreased in ZSS- compared to X-ALD patients (Figure 2, Figure 3 and Figure 4; Table A1). 

A lack of total ether-phospholipids was also reported by Herzog et al. [16] as an outcome of a global metabolomic study on peroxisomal biomarkers. Plasmalogens have been established for a long time as classical markers for the diagnosis of ZSS, with research showing the diagnostic potential as early as 1983 [21]. Our kit includes 38 acyl-ethyl-linked phospholipids of which the majority were found to be clearly reduced in their plasma levels compared to controls and X-ALD samples (Table A1). Plasmalogen levels are low or undetectable in ZSS patients, as biosynthesis starts in the peroxisome by acyldihydroxyacetonephosphate (acylDHAP) formation using dihydroxy-acetonephosphate acyltransferase (DHAPAT), and then introducing the ether-bond by alkyldihydroxyacetonephosphate synthase (ADHAPS) generating alkylDHAP. Both DHAPAT and ADHAPS are strictly peroxisomal in contrast to other enzymes needed for plasmalogen synthesis [22,23]. Interestingly, we found that certain specific ether-lipids—as well as the total sum of plasmalogen-candidates (phophatidylcholine-plasmalogens)—are unexpectedly mildly increased in X-ALD, compared to controls. We did not find any published data concerning the regulation of ether-phosphatidylcholines analyzed from serum in this study. We theorize, that due to the peripheral lack of ether-lipids in tissue, more ether-lipids are mobilized in the blood to compensate for the destruction in tissues. However, using this dataset, we are not able to give definitive conclusions and this should be analyzed in further studies.

Due to their low plasma concentrations, plasmalogens are classically measured from erythrocyte membranes by gas chromatography coupled mass spectrometry [19]. However, red blood cell membrane analysis can be cumbersome, as membranes need to be freshly prepared from whole blood before hemolysis. As whole blood cannot be stored for a prolonged time, retrospective analysis from samples from biobanks is hindered. Since the kit is sensitive enough for the conclusive determination of included ether-phospholipid levels from patient serum, it provides a promising option for the laboratory plasmalogen diagnostic if no whole blood samples are available. Of particular importance here is that the plasmalogen markers C18-diacetal (four patients) and C16-diacetal (three patients) determined by GC-MS were within the normal reference range, whereas the plasmalogen marker PC ae C36:4 was significantly reduced in these patients and thus may enable more reliable differentiation (Table A2).

In our analyses, we did also find a correlation between the GC-MS plasmalogen marker C18-diacetal with the corresponding ether-phosphatidylcholines PC ae C36:0 and C36:4. However, we could only include 5 patients in the correlation analysis as most ZSS samples did not contain C18-diacetal in levels high enough for quantification. Being a parameter analyzed by FIA-MS, PC ae C36:4 is a high throughput-able parameter and can also be analyzed without perilous and manual labor-intensive derivatization necessary in GC-MS.

Most sphingomyelins (SM C16:0, C18:1, C16:1, C18:0, C24:0, C24:1) are also significantly decreased in ZSS patients, but not in X-ALD (Figure 3 and Figure 4, Table A3). In ZSS patients, sphingomyelins such as SM C16:0, C18:1, C16:1, C18:0, C24:0, C24:1 have been described to be decreased in various lipidomics studies [14,16]. In contrast, sphingomyelin C26:0, a VLCFA-sphingomyelin, is significantly increased in both peroxisomal disorders. Possibly, this is the result of an overrepresentation of VLCFAs due to limited degradation in peroxisomes. The attachment of the second acyl-chain is occurring due to action of an alkyl/acyl-GPA acyltransferase in the endoplasmatic reticulum [24] or more probably remodeling in Lands’ cycle [25]. Unfortunately, not much is known about enzymes acyl-specificity and regulation for VLCFAs. Similarly, this can also be observed for sphingomyelins.

Among the discussed markers which are linked to an VLCFA is also lysoPC C26:0 which has been previously described [20] and is used as first-tier newborn screening marker for X-ALD [26].

Using the commercial kit, we may also be able to differentiate two patients of another type of peroxisomal disorder, D-BPIII, using a small set of markers from ZSS and X-ALD patients. D-BP is a central protein for the degradation of VLCFA in peroxisomes, therefore accumulations of VLCFA as in ZSS and X-ALD are generally observed. Thus, an alteration of biomarkers in the commercial kit that relates to VLCFAs such as lysoPC 26:0 and SM 26:0 as in ZSS and X-ALD is only consistent and were also found by other lipidomics studies [15]. Additionally, a drop in the plasmalogen markers C16- and C18-diacetal was found in GC-MS analysis of one patient, complicating a diagnosis of those patients purely based on GC-MS data. While a drop of PC ae C36:4 compared to the control group was also found, the alteration is considerably milder compared to typical ZSS patients. Albeit the small sample size (*n* = 2) is prohibitive for making further assumptions, a small alteration of this lipid class may originate from a general disturbance in lipid homeostasis, due to impaired VLCFA metabolism. Similarly, to our results in serum, as study by Herzog et al. [17] found similar alterations in patient fibroblasts. Thus, the proposed set of markers may provide an alternative identification of those disorders, albeit these results should be confirmed in further, larger, studies.

As expected, using clustering techniques, the profile of the peroxisomal single protein disorder X-ALD (Figure A2) is not as aberrant as the peroxisomal biogenesis disorder ZSS (Figure A1), as ZSS impairs numerous peroxisomal metabolic pathways. However, using the proposed set of markers (e.g., lysoPC C26:0, SM C26:0) we were able to differentiate patients from controls in most cases (Figure 3). Yet, when using lysoPC C26:0 as only marker, there is a considerable overlap between controls and X-ALD patients. Similarly, to the method in this commercial kit, a study by Natarajan et al. showed that utilizing flow injection mass spectrometry (FIA-MS), lysoPC C26:0 was overestimated especially in the lower concentration range [27]. Thus, maybe leading to impaired separation of controls and patients also in this commercial kit.

Due to the principles of applied analysis, it is not possible to specify exact acyl-chains in measured lipids. The identification primarily occurs by the mass of the molecular ion (head group and acyl chains) and the fragment of the group-specific head-group-fragment. This method therefore cannot separate between analytes with the same total carbon chain length or double bonds (same mass, isobaric analytes). Additionally, the specific positioning, sn-1 or sn-2, of fatty acids cannot be differentiated, albeit these can have major implications for functions of those lipids. Thus, after a positive screening result, specific findings should be confirmed using alternative methods. As such, this is a common caveat in lipidomics owing to the high complexity of the lipidome, and acyl-chains are rarely directly identified to be specific (e.g., specific double bond position) fatty acids. Bearing this in mind however, the simplification of the lipidome creates more easily interpretable data, allowing for higher throughput especially in non-metabolomics dedicated facilities.

To conclude, using the Biocrates AbsoluteIDQ p180-kit we were able to reproduce some central, diagnostically relevant findings from untargeted metabolomics studies on new biomarkers for peroxisomal disorders. Especially PC ae C36:4 as marker for plasmalogens in plasma may provide advanced insights and enables also retrospective analyses from biobank samples.

Thus, the kit may be applied for an expanded laboratory diagnostic for patients with a potentially unclear peroxisomal disorder or related disease. However, in case of unusual metabolic findings, the high efforts to perform a global untargeted metabolomic or genetic follow-up study can be well justified.

## 4. Materials and Methods

### 4.1. Study Cohort

A total of 17 ZSS patients with a mutation in the Peroxisome biogenesis factor 1 (*PEX1*) gene with a mean age of 2.6 ± 4.9 years was analyzed. Patients had a ratio of lignoceric acid (C22:0) to behenic acid (C24:0) of 1.6 ±0.3 as determined by total serum fatty acid analysis by GC-MS.

In addition to these peroxisomal biogenesis defects patients, a total of 38 patients with peroxisomal single enzyme defects were examined with this kit. This group consisted of 36 X-ALD patients with mutations in the *ABCD1* gene and 2 patients with D-bifunctional protein deficiency type III (D-BPIII) with mutations in the *HSD17B4* gene.

The control cohort consisted of 28 age-matched patients, which submitted serum for a routine check-up of amino acids in serum, but did not display any abnormalities and did not have any prior history of peroxisomal disorders.

All procedures were in accordance with the ethical standards of the responsible committee on human experimentation and with the Helsinki Declaration. The institutional Ethics Committee of University Medical Centre Göttingen (UMG) approved this study (protocol code 4/11/16, date: 6 December 2016).

### 4.2. Metabolomics Analysis

Metabolomics analysis was performed using the AbsoluteIDQ p180-Kit (Biocrates Lifesciences, Innsbruck, Austria) applied on to a BEH Amide column (Waters, Milford MA, USA) and a Xevo TQ-S mass spectrometer (Waters, Milford, MA, USA). The kit was processed in accordance with the guidelines of the manufacturer. Measurement of the kit consists of two separate analyses, flow injection analysis (FIA-MS) for lipids and liquid chromatography MS (LC-MS) for amines. FIA-MS is used for quantification of up to 145 metabolites (including 40 acyl-carnitines, 15 sphingolipids, 76 phosphatidylcholines, and 15 lysophosphatidylcholines). LC-MS analysis includes measurement of 21 canonical amino acids and 21 other biogenic amines, such as neurotransmitters. The kit is set up in a 96-well scheme and utilizes a seven-point calibration curve for amines and a one-point calibration for lipids, as well a proprietary deuterated internal standard mixture for each sample. Every kit measurement includes a three-level quality control (QC) prepared from human serum, of which the medium level (QC2) is repeated four times across the kit plate.

Briefly, 10 µL of patient serum was applied to the kit plate, which had been lined with an internal standard solution and dried under a nitrogen pressure manifold (Waters, Milford). Consecutively, the dried sample plate was treated with phenyl isothiocyanate to derivatize amines and dried under nitrogen flow.

Samples from the plate were extracted using 5 mM ammonium acetate in methanol and diluted in flow injection analysis solvent for FIA analysis and water for LC-MS analysis, respectively. FIA and LC-MS runs were executed successively. LC-MS data were first analyzed using MassLynx V4.1 software (Waters, Milford, MA, USA) using the supplied analysis method by Biocrates, confirmed manually, and imported into the software supplied by the kit (MetIDQ Carbon, Biocrates, Innsbruck, Austria). FIA data were directly loaded into MetIDQ and interpreted automatically.

Validation of the kit run was done using supplied quality control (QC) samples and MetIDQ software.

### 4.3. Denotation of the Lipid Classes

Lipids are named according to the specifications of the manufacturer but oriented on the recommendation of the international lipid classification and nomenclature committee [28]. Summarizing, lipids are denoted by their specific class/headgroup (PC: phosphatidylcholines, LPC: lysophosphatidylcholines, SM: sphingomyelins; C (without additions) carnitines). Type of bonding of fatty acids with the group is denoted as “a” for acyl-bonds and “e” for ether-bonds.

Total amount of carbon atoms inside all fatty acid chains is summed and displayed as number, separates with a “:” to indicate the total number of double bonds/unsaturation.

### 4.4. Statistical Procedures

Data of metabolites were exported from MetIDQ as concentration data and metabolites with a concentration above the lower limit of quantification were subjected to statistical analysis using MetaboAnalyst 4.0 [29]. Data were normalized using cube root transformation and auto scaling.

Volcano plots were generated using Python 3.6 and Matplotlib 3.0 [30], significant results with an FDR below 0.05 and a log2 fold change greater than 1.0 or lower than −1.0 are displayed. Hierarchical clustering analysis was performed on normalized data of the 65 metabolites with the lowest *p*-values after Student’s *t*-test using Euclidean distance measure and Ward clustering algorithm.

Data from single metabolites was analyzed using Prism 8.3 (GraphPad, San Diego, CA, USA). If not otherwise mentioned, data is shown as mean ± SEM.

Significance testing was done using one-way ANOVA multiple comparisons analysis with Tukey correction and significance levels are displayed as * *p* < 0.05, ** *p* < 0.01, *** *p* < 0.001.

## Data Availability

Data is contained in the article.

## Appendix A

**Table A1:** Serum from patients analyzed using the Biocrates AbsoluteIDQ p180 kit. ZSS patients show aberrant serum concentration of ether-linked phospholipids. Concentrations of ether-phosphatidylcholine species (PC ae) in µM, *n* = 36 (X-ALD/*ABCD1*), 28 (Controls), 17 (ZSS/*PEX1*).

	Mean Serum Concentration (µM)			SEM (µM)		
	X-ALD	Control	ZSS	X-ALD	Control	ZSS
PC ae C30:0	0.51	0.32	0.28	0.04	0.03	0.04
PC ae C30:1	0.11	0.07	0.08	0.02	0.01	0.01
PC ae C30:2	0.36	0.16	0.11	0.03	0.02	0.02
PC ae C32:1	1.71	1.58	0.40	0.09	0.12	0.06
PC ae C32:2	0.54	0.40	0.16	0.02	0.03	0.02
PC ae C34:0	1.12	1.04	0.91	0.05	0.14	0.12
PC ae C34:1	6.33	6.32	2.08	0.33	0.56	0.32
PC ae C34:2	6.94	5.52	1.04	0.43	0.38	0.20
PC ae C34:3	4.23	3.66	0.60	0.26	0.32	0.14
PC ae C36:0	1.29	0.67	1.05	0.10	0.07	0.14
PC ae C36:1	65.55	24.81	30.94	5.74	2.99	6.59
PC ae C36:2	19.58	8.30	6.12	1.55	0.63	1.16
PC ae C36:3	4.13	3.80	0.75	0.19	0.28	0.12
PC ae C36:4	7.76	9.28	0.67	0.70	1.04	0.20
PC ae C36:5	4.44	5.64	0.53	0.34	0.67	0.13
PC ae C38:0	1.69	0.80	0.43	0.12	0.06	0.06
PC ae C38:1	27.12	7.58	18.46	2.67	1.72	4.69
PC ae C38:2	22.05	6.94	11.71	2.32	1.39	2.60
PC ae C38:3	21.39	10.59	7.20	1.43	1.41	1.16
PC ae C38:4	8.51	8.36	1.63	0.35	0.71	0.30
PC ae C38:5	5.81	8.58	0.59	0.46	0.95	0.14
PC ae C38:6	1.90	2.72	0.25	0.15	0.30	0.04
PC ae C40:1	3.83	1.34	1.00	0.27	0.17	0.20
PC ae C40:2	6.12	3.28	2.88	0.38	0.40	0.42
PC ae C40:3	10.15	5.39	4.19	0.62	0.92	0.62
PC ae C40:4	5.30	3.38	1.61	0.25	0.38	0.32
PC ae C40:5	6.09	4.73	1.30	0.43	0.43	0.21
PC ae C40:6	1.95	2.34	0.32	0.12	0.21	0.05
PC ae C42:0	1.63	0.66	0.72	0.09	0.05	0.08
PC ae C42:1	2.84	0.82	0.79	0.17	0.14	0.15
PC ae C42:2	2.23	0.82	0.56	0.13	0.11	0.09
PC ae C42:3	2.37	1.01	0.67	0.15	0.13	0.12
PC ae C42:4	1.65	1.03	0.48	0.08	0.09	0.08
PC ae C42:5	2.69	2.24	0.98	0.13	0.16	0.09
PC ae C44:3	0.81	0.30	0.26	0.05	0.05	0.05
PC ae C44:4	0.80	0.43	0.23	0.04	0.03	0.03
PC ae C44:5	1.19	1.07	0.22	0.06	0.08	0.03
PC ae C44:6	0.74	0.68	0.22	0.04	0.05	0.03

**Table A2:** ZSS patients with irregularly high GC-MS plasmalogen markers can still be identified as plasmalogen deficient by the FIA-MS marker PC ae 36:4. Red blood cell membrane plasmalogen from *PEX1* patients was analyzed using GC-MS and serum from the same sample was analyzed using the Biocrates AbsoluteIDQ p180 kit. For reference range 95% confidence interval from 28 controls were used.

	C16:0-Diacetal (Ratio to 16:0 FA)		C18:0-Diacetal (Ratio to 18:0 FA)		PC ae 36:4 (µM)	
Patient	Subject	Reference Range	Subject	Reference Range	Subject	95% Confidence
ZSS_1	5.2	6.8–11.9	11.2	10.6–24.9	0.801	7.24–11.32
ZSS_2	9.0	6.8–11.9	20.4	10.6–24.9	0.619	7.24–11.32
ZSS_3	6.9	6.8–11.9	15.4	10.6–24.9	3.69	7.24–11.32
ZSS_4	7.3	6.8–11.9	18.0	10.6–24.9	1.25	7.24–11.32

**Table A3:** VLCFA sphingomyelin species are elevated in serum of X-ALD and ZSS patients, non-VLCFA sphingomyelin species are reduced in ZSS patient compared to controls. Serum from patients analyzed using the Biocrates AbsoluteIDQ p180 kit. Concentrations of sphingomyelin species (SM) in µM, *n* = 36 (X-ALD/ABCD1), 28 (Controls), 17 (ZSS/*PEX1*).

	Mean Serum Concentration (µM)			SEM (µM)		
	X-ALD	Control	ZSS	X-ALD	Control	ZSS
SM C16:0	113.98	131.31	62.73	4.44	14.44	5.68
SM C16:1	15.72	15.41	6.61	0.57	1.12	0.63
SM C18:0	14.70	29.81	7.93	0.72	4.22	1.19
SM C18:1	8.35	13.57	4.23	0.44	1.34	0.75
SM C20:2	0.48	0.56	0.19	0.04	0.13	0.03
SM C24:0	33.97	28.31	13.39	1.46	2.15	1.28
SM C24:1	59.63	68.97	30.85	2.79	6.11	3.14
SM C26:0	0.84	0.28	0.81	0.07	0.03	0.14
SM C26:1	1.27	0.56	1.45	0.09	0.06	0.29

**Table A4:** Lysophosphatidylcholine species in serum of X-ALD and ZSS patients are elevated compared to controls. Serum from patients analyzed using the Biocrates AbsoluteIDQ p180 kit. Concentrations of lysophosphatidylcholine species (lysoPC) in µM, *n* = 36 (X-ALD/*ABCD1*), 28 (Controls), 17 (ZSS/*PEX1*).

	Mean Serum Concentration (µM)			SEM (µM)		
	X-ALD	Control	ZSS	X-ALD	Control	ZSS
lysoPC C16:0	205.33	189.41	120.50	11.87	14.50	15.53
lysoPC C16:1	5.03	3.15	2.09	0.37	0.29	0.23
lysoPC C17:0	4.16	4.49	1.80	0.30	0.46	0.34
lysoPC C18:0	65.24	78.80	43.94	3.78	9.05	7.19
lysoPC C18:1	37.07	36.46	24.78	1.84	4.98	3.50
lysoPC C18:2	38.34	21.52	16.68	2.00	2.50	2.78
lysoPC C20:3	2.90	3.46	1.82	0.15	1.03	0.26
lysoPC C20:4	7.06	6.79	3.26	0.32	1.23	0.46
lysoPC C24:0	0.99	0.34	0.69	0.06	0.04	0.09
lysoPC C26:0	2.12	0.54	1.29	0.15	0.11	0.16
lysoPC C26:1	0.39	0.19	0.43	0.02	0.03	0.05
lysoPC C28:0	1.61	0.56	0.75	0.14	0.10	0.13
lysoPC C28:1	1.11	0.49	0.48	0.08	0.07	0.07

**Table A5:** D-BPIII patient with irregularly low GC-MS plasmalogen markers can still be identified as different to ZSS patients, but also controls, by the FIA-MS marker PC ae 36:4. Red blood cell membrane plasmalogen from D-BPIII patient was analyzed using GC-MS and serum from the same sample was analyzed using the Biocrates AbsoluteIDQ p180 kit. For reference range the 95% confidence interval from 28 controls was used.

	C16:0-Diacetal (Ratio to 16:0 FA)		C18:0-Diacetal (Ratio to 18:0 FA)		PC ae 36:4 (µM)	
Patient	Subject	Reference Range	Subject	Reference Range	Subject	95% Confidence
DBIII_1	4.3	6.8–11.9	6.3	10.6–24.9	5.32	7.24–11.32
DBIII_2	12.8	6.8–11.9	15.0	10.6–24.9	2.82	7.24–11.32
